# Supplementary material for: The intensities of canonical senescence biomarkers integrate the duration of cell-cycle withdrawal
Source: Nat Commun. 2023 Jul 27;14:4527. doi: 10.1038/s41467-023-40132-0 (PMC10374620; doi:10.1038/s41467-023-40132-0)
Supplement: Supplementary file 1 — Supplementary Information [file 41467_2023_40132_MOESM1_ESM.pdf]

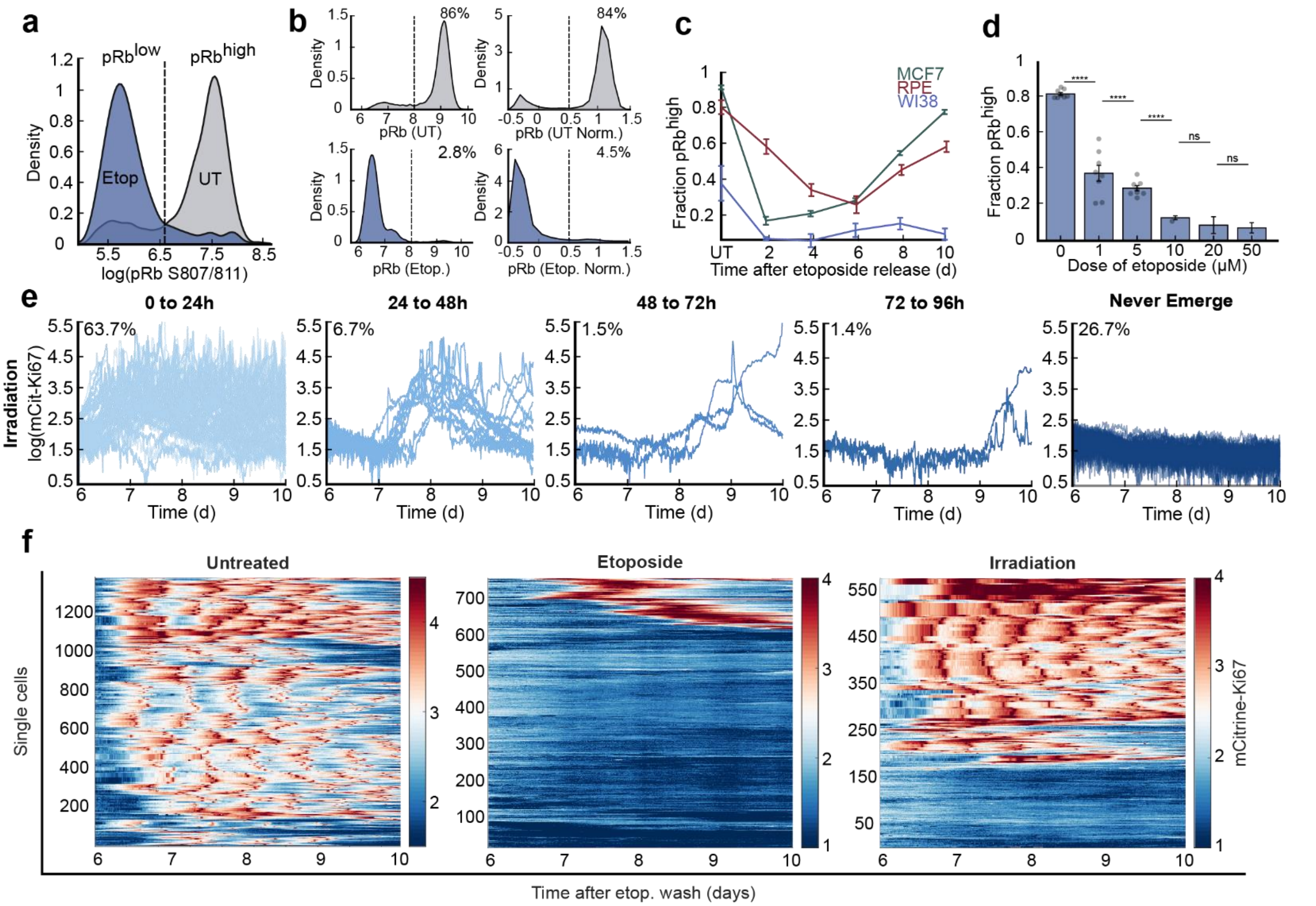

Supplemental Figure 1. Release from acute DNA damage stress induces population heterogeneity in cell cycle fate. (a) Definition of phospho-Rb^{low} versus phospho-Rb^{high} in MCF10A cells released for 6d from a 24h treatment of 10 μM etoposide. UT, untreated. (b) The fraction of cells classified as phospho-Rb^{high} (using anti-pRb S807-811) with and without normalization to total Rb is similar. (c) MCF7, RPE-hTERT, and WI38-hTERT cells were treated with 10 μM etoposide for 24h before being washed and allowed to recover for up to 10d. (d) MCF10A cells were treated with increasing concentrations of etoposide for 24h before being fixed and stained for phospho-Rb at 6d after release from drug. (e) MCF10A cells expressing endogenously tagged mCitrine-Ki67 were plated on day 0 and treated with 10 Gy ionizing radiation the following day. On day 5, Ki67^{off} cells were isolated by flow cytometry, plated, and allowed to grow for 24h before being imaged for 96h by time-lapse microscopy. Single-cell traces are grouped based on their relative timing of cell-cycle re-entry from the Ki67^{off} state and the percentage of cells in each group is indicated. 200 traces are plotted in total. (f) Heatmaps of all traces from time-lapse microscopy for untreated, etoposide-released, and irradiated cells from **Fig. 1e and Supplementary Fig. 1e.**

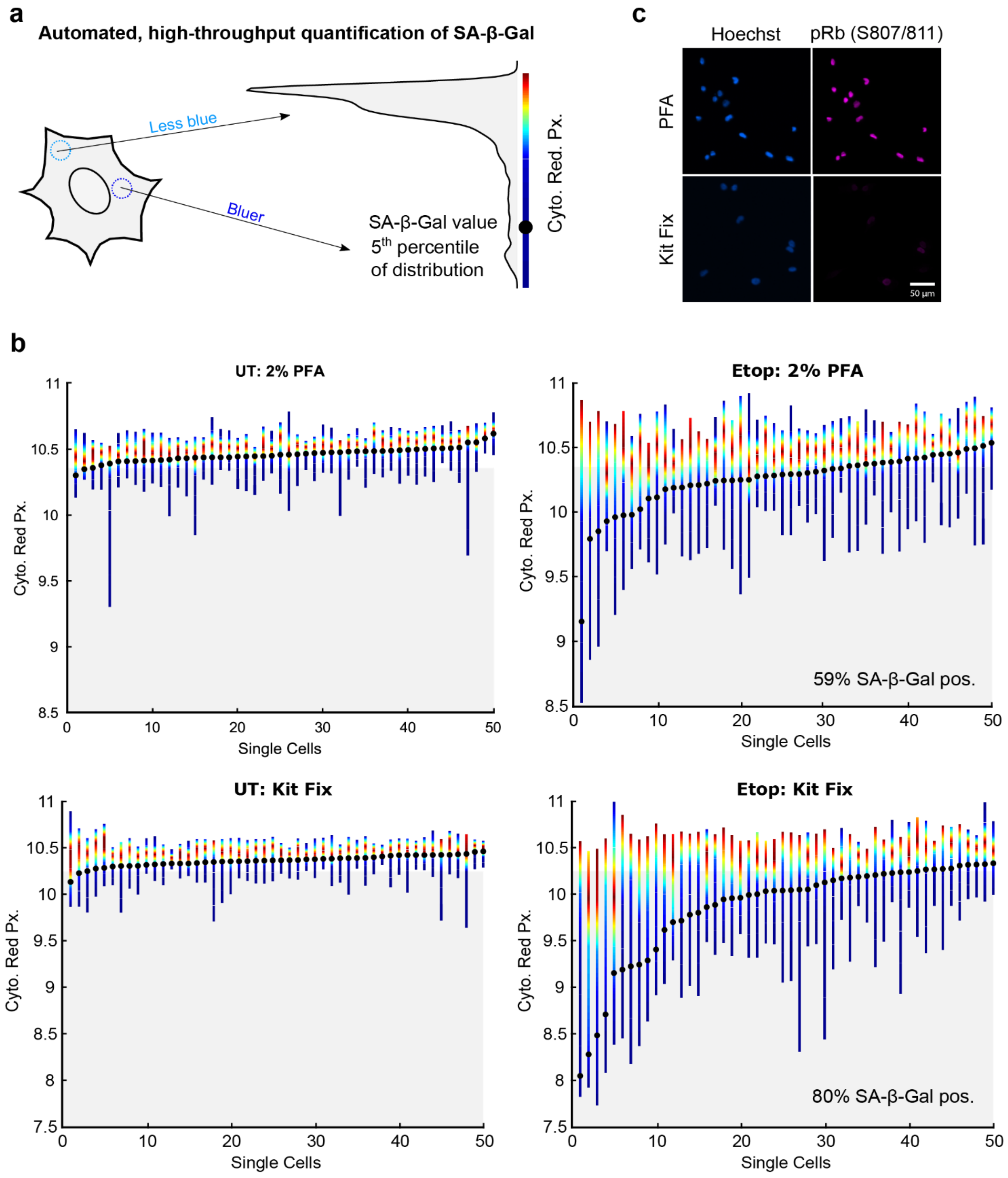

Supplemental Figure 2. Automated, high-throughput quantification of the SA-β-Gal stain in single cells induced to senescence. (a) A single representative cell's cytoplasmic red pixel distribution. The heatmap corresponds to the relative frequency of events along the distribution. (b) Quantification of SA-β-Gal for 50 single cells left untreated or released for 6d from a 24h treatment with 10 µM etoposide, for both 2% PFA (which is optimal for immunofluorescence) and the CST kit fixative (which is optimal for detection of SA-β-Gal). The black dot is the value at the 5th percentile of the distribution, which is the SA-β-Gal score for that cell. The percentage of SA-β-Gal^{pos} cells was calculated using the 95th percentile of all untreated cells as the cutoff. Heatmap coloring as in panel A. (c) Comparison of immunofluorescence for phospho-Rb (S807/811) after fixation with 2% PFA versus CST SA-β-Gal kit fixative in WT MCF10A cells showing the weaker immunofluorescence signal with the CST kit fixative.

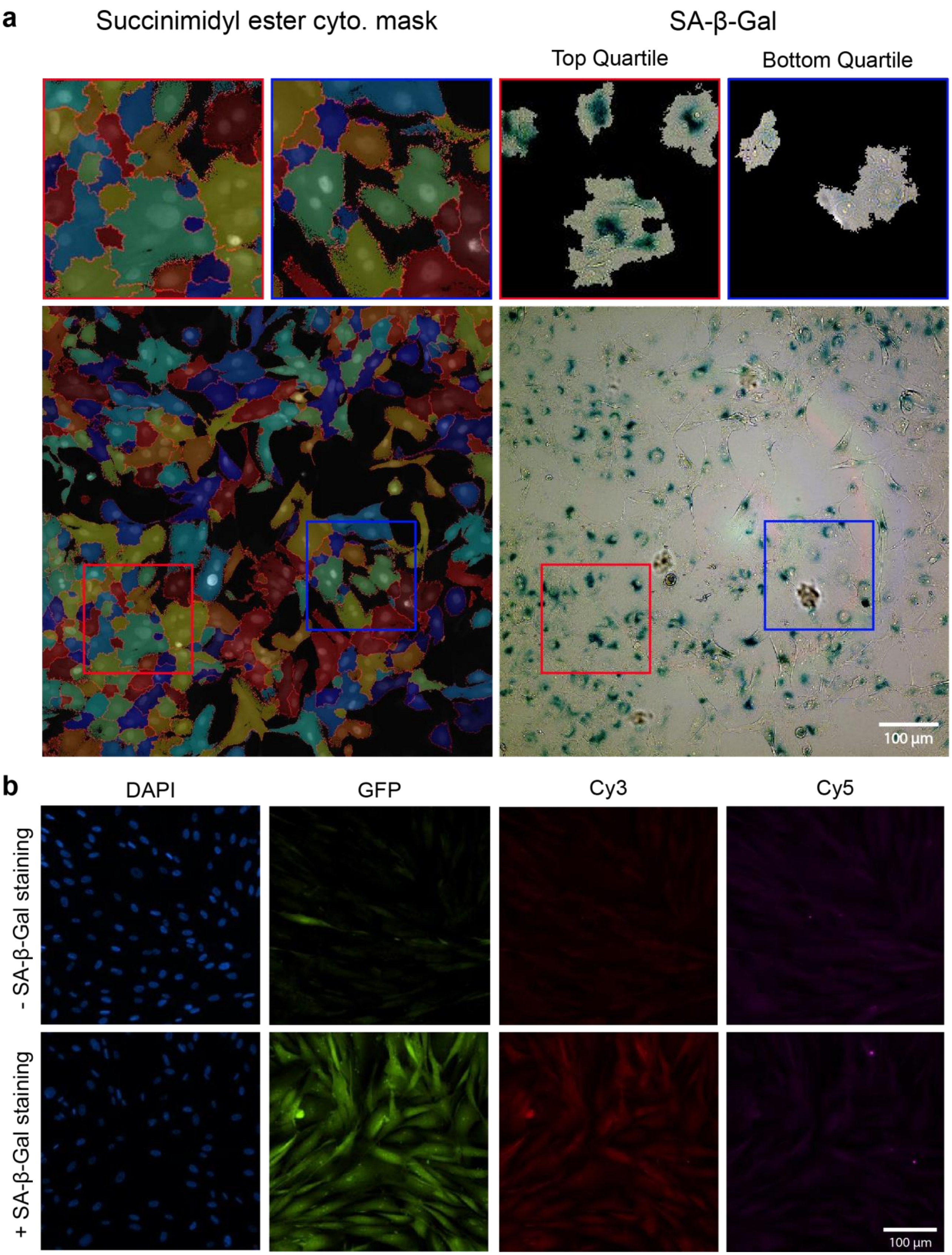

Supplemental Figure 3. Validation of the SA-β-Gal quantification method and comparison of background fluorescence. (a) Validation of whole-cell segmentation using the succinimidyl ester stain and SA-β-Gal quantification by displaying the upper and lower quartiles of SA-β-Gal signals from the binary cytoplasmic mask after a 4d release from a 24h pulse of 10 µM etoposide in MCF10A cells. (b) Comparison of background fluorescence in each fluorescent channel with and without co-staining WI38 cells for SA-β-Gal (kit fixative).

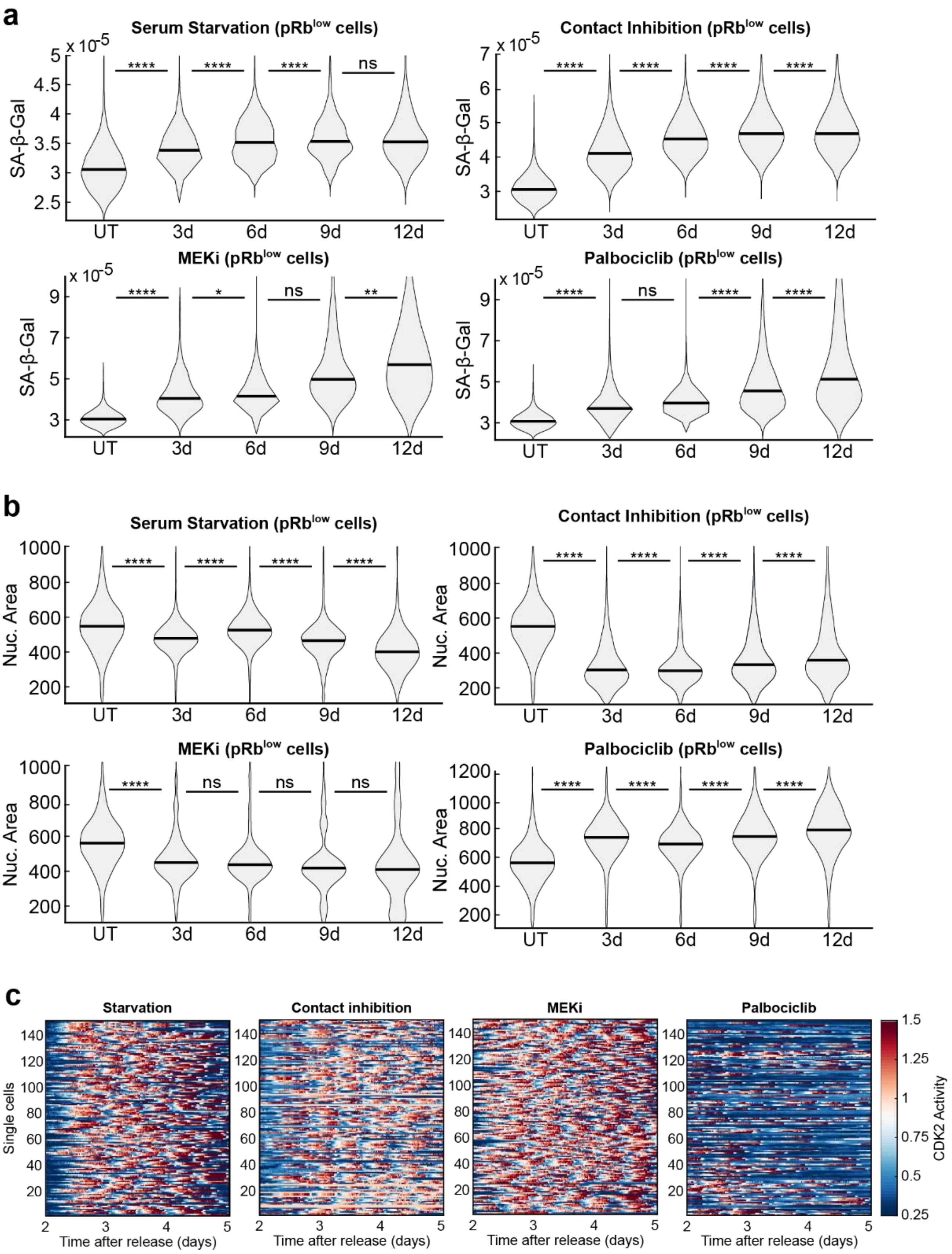

Supplemental Figure 4. SA-β-Gal staining, nuclear area, and cell-cycle re-entry after increasing durations of quiescence induction. (a) Violin plots of the data plotted in **Fig. 3e** and their (b) corresponding nuclear areas for pRb^{low} cells. (a-b) Black lines are the median of each distribution. (c) Heatmaps of 150 representative single-cell traces of CDK2 activity for cells released from 2 weeks of each quiescence-inducing treatment.

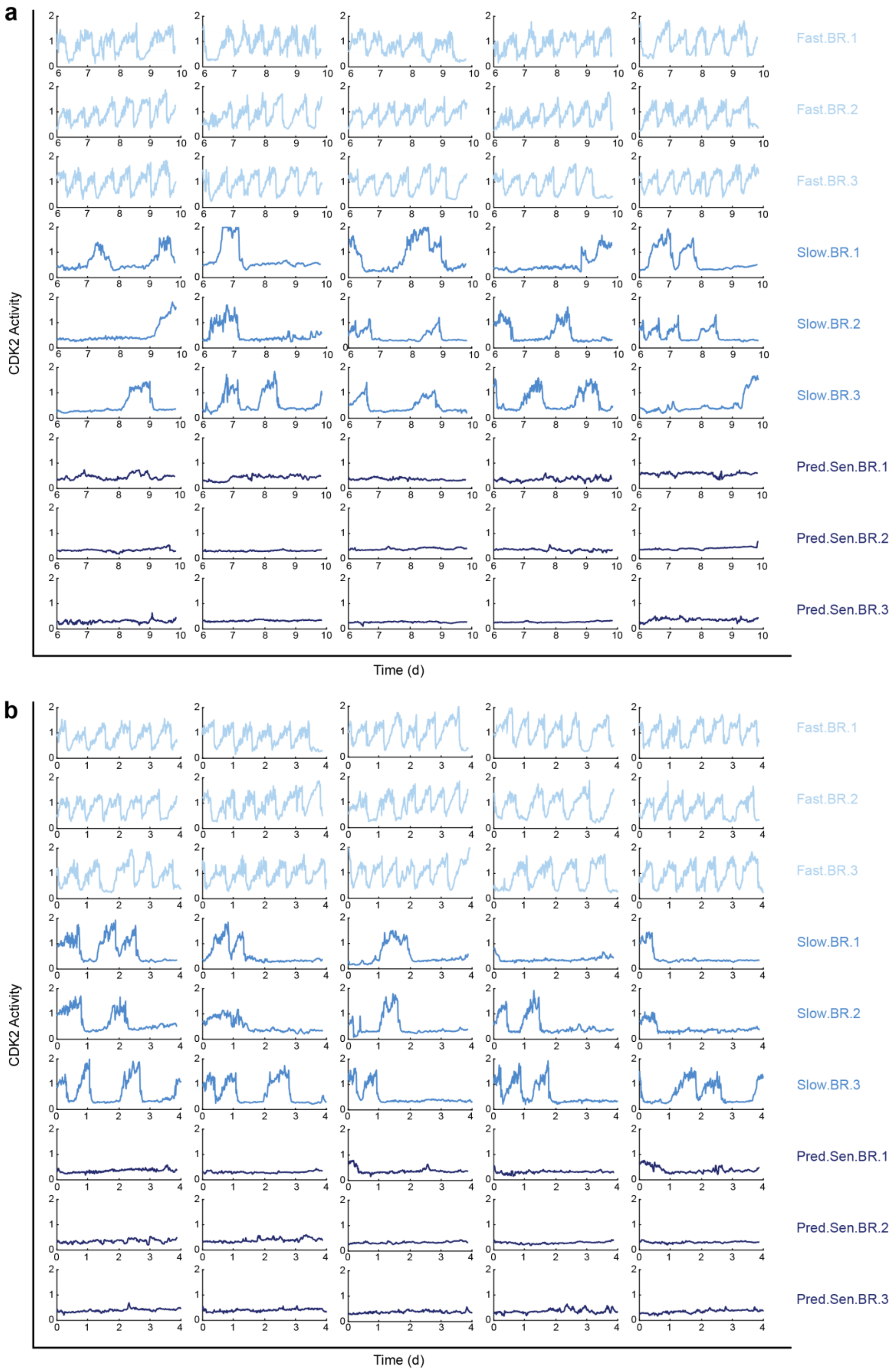

Supplemental Figure 5. Representative single-cell traces from three biological replicates for etoposide and hydrogen peroxide release. (a-b) Five representative traces across three independent biological replicates (BRs) for fast-cycling, slow-cycling, and predicted-senescent categories following release from a 24h treatment with 10 μM etoposide and filmed from day 6-10 after release (a) or following release from a 2h treatment with 100μM hydrogen peroxide and filmed for days 0-4 after release (b).

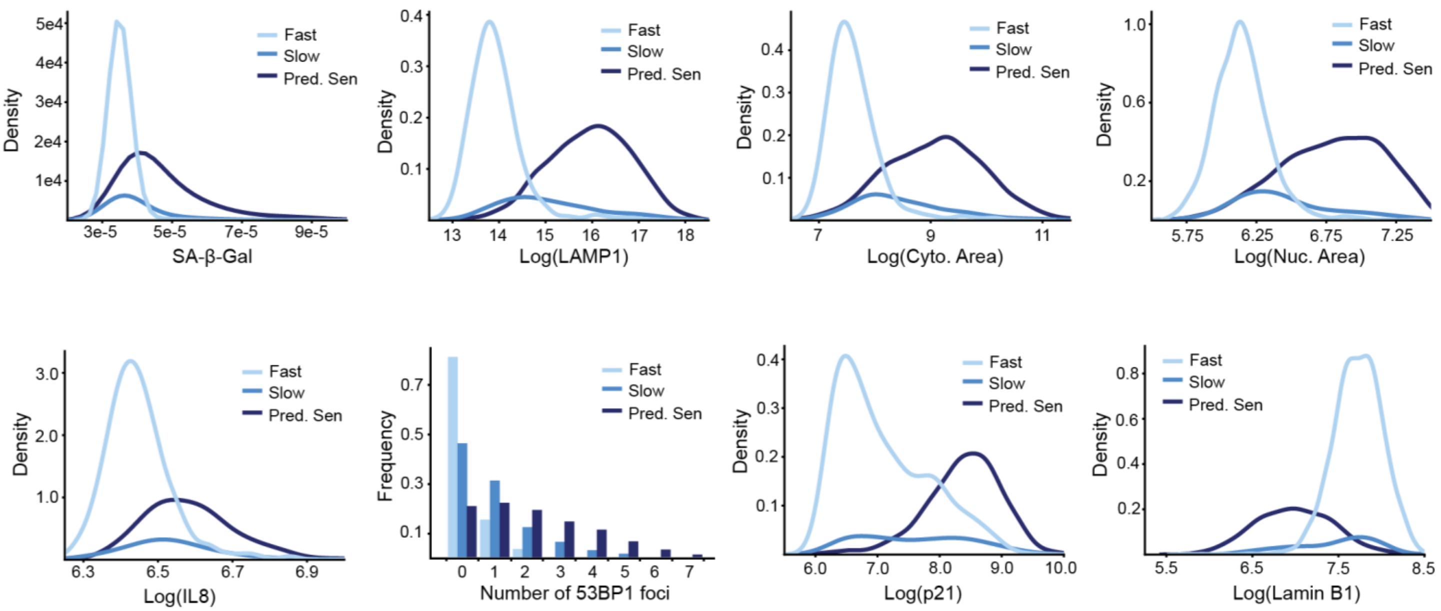

Supplemental Figure 6. Distributions of senescence marker intensity for fast-cycling, slow-cycling, and predicted-senescent cells. Probability density estimates for data plotted in **Figure 4**. Cells were categorized as fast-cycling, slow-cycling, or predicted-senescent and the marker intensity distribution for every marker was plotted. Number of 53BP1 foci for each cell is an integer and thus is plotted as a bar histogram.

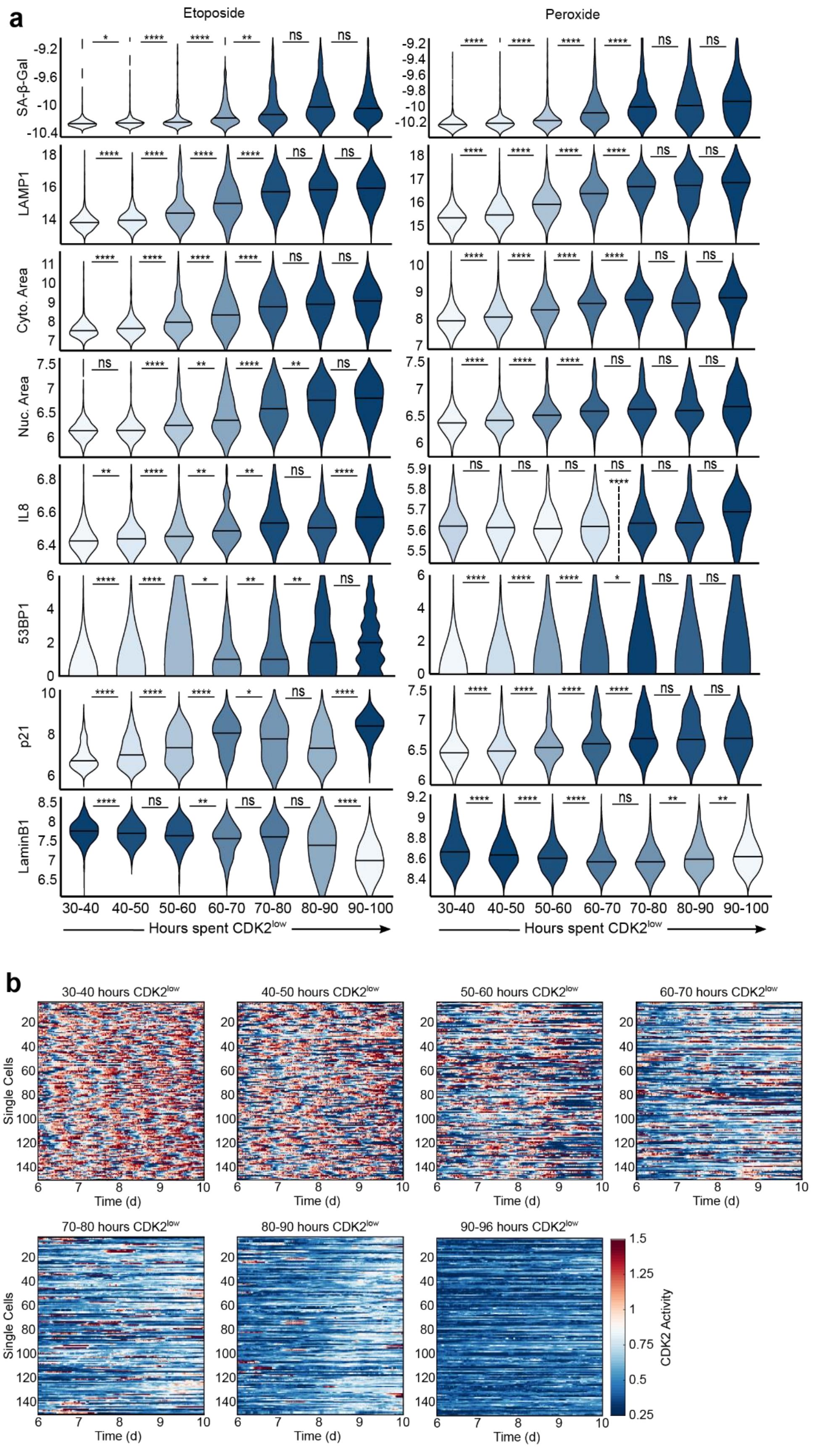

Supplemental Figure 7. Distributions of senescence marker intensity with respect to cell-cycle status. (a) Violin plots of the data plotted in **Figure 5**. Black lines are the median of each distribution. An additional statistical test was performed on peroxide released cells stained for IL8 for cells that spent 30-70 hours CDK2^{low} compared to cells that spent 70-96 hours CDK2^{low}, as indicated by the dashed line. (b) Heatmaps of 148 representative single-cell traces of CDK2 activity for each time bin specified in **Supplementary Fig. 7a**.

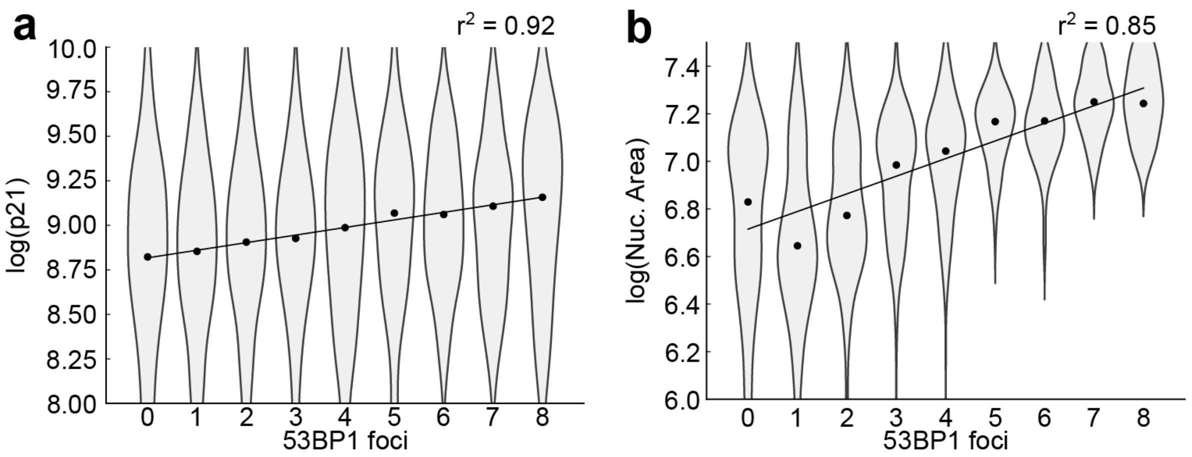

Supplemental Figure 8. p21 and nuclear area binned by 53BP1 foci number. (a-b) MCF10A cells were released for 6d from a 24h treatment of 10 μM etoposide and then fixed and stained for Hoechst, p21, and 53BP1. The distributions of p21 (a) and nuclear area (b) are plotted for increasing numbers of 53BP1 nuclear bodies. Black dots represent the mean of each distribution. The line is the line of best fit along the means.

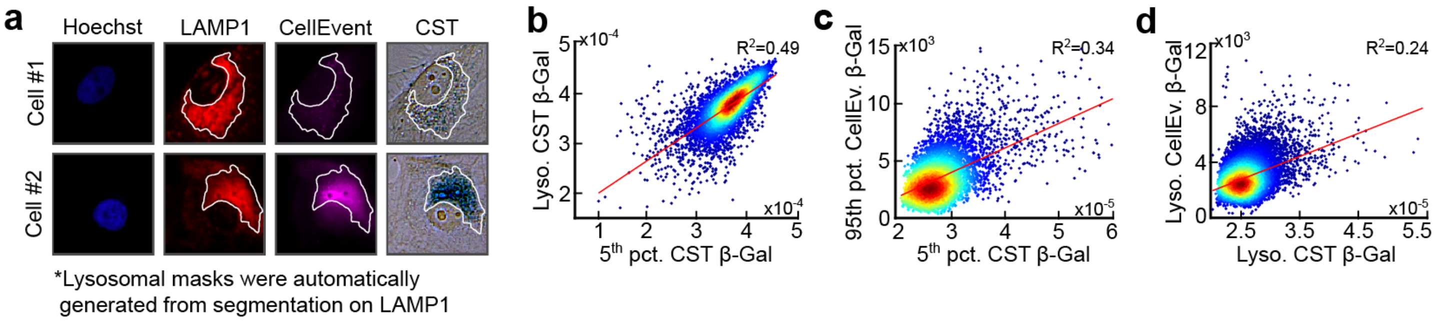

Supplemental Figure 9. CST SA-β-Gal, Cell Event SA-β-Gal, and LAMP1 multiplexing. (a) MCF10A cells were co-stained for LAMP1, the gold-standard colorimetric SA-β-Gal kit from CST used throughout this work, and the newer fluorescent Cell Event SA-β-Gal kit 6d after release from a 24h treatment of 10 μM etoposide. Lysosomal masks were automatically generated from thresholding on LAMP1 staining. (b) The mean lysosomal signal of CST SA-β-Gal from LAMP1 segmentation versus the 5th percentile quantification of CST SA-β-Gal used throughout this work. (c) The 95th percentile quantification of Cell Event SA-β-Gal versus the 5th percentile quantification of CST SA-β-Gal, showing the level of agreement between these two kits. (d) The mean lysosomal CST SA-β-Gal versus the mean lysosomal Cell Event SA-β-Gal from LAMP1 segmentation, showing the level of agreement between these two kits.
