# Supplementary material for: The intensities of canonical senescence biomarkers integrate the duration of cell-cycle withdrawal
Source: Nat Commun. 2023 Jul 27;14:4527. doi: 10.1038/s41467-023-40132-0 (PMC10374620; doi:10.1038/s41467-023-40132-0)
Supplement: Supplementary file 4 — Description of Additional Supplementary Files [file 41467_2023_40132_MOESM4_ESM.pdf]

Supplementary Movie 1. Senescence biomarker intensities reflect cell-cycle histories for fast-cycling, slow-cycling, and predicted-senescent cells. Representative fast-cycling (left), slow-cycling (middle), and predicted-senescent (right) cells for the experiment in **Fig. 4**. Fixed-cell staining at the final movie frame for Hoechst, LAMP1, succinimidyl ester 488, 53BP1, and SA-β-Gal are shown.

Supplementary Data 1. Number of replicates, sample size, and statistical analysis for each experiment.
